# Indications, success, and adverse event rates of pediatric endoscopic retrograde cholangiopancreatography (ERCP): a systematic review and meta-analysis

**DOI:** 10.1186/s12887-023-04392-5

**Published:** 2023-11-24

**Authors:** Amirhossein Hosseini, Mohammad Hassan Sohouli, Elham Sharifi, Aliakbar Sayyari, Kannan Sridharan, Saleheh Tajalli, Negar Imanzadeh, Somaye Fatahi

**Affiliations:** 1 Pediatric Gastroenterology, Hepatology, and Nutrition Research Center, Research Institute for Children’s Health, Shahid Beheshti University of Medical Sciences, Tehran, Iran; 2 Student Research Committee, Department of Clinical Nutrition and Dietetics, Faculty of Nutrition and Food Technology, Shahid Beheshti University of Medical Sciences, Tehran, Iran; 3 Department of Nutrition, School of Public Health, Iran University of Medical Sciences, Tehran, Iran; 4 Department of Pharmacology and Therapeutics, College of Medicine and Medical Sciences, Arabian Gulf University, Manama, Bahrain; 5 School of pharmacy, Shahid Beheshti University of Medical Sciences, Tehran, Iran; 6 Department of Clinical Nutrition and Dietetics, Faculty of Nutrition and Food Technology, Shahid Beheshti University of Medical Sciences, Tehran, Iran

**Keywords:** Endoscopic retrograde cholangiopancreatography, Success, Safety, Children, Adolescents, Meta-analysis

## Abstract

**Background:**

To improve knowledge on endoscopic retrograde cholangiopancreatography (ERCP) in children, we aimed to study the proportion of indications, success rate and complication of ERCP.

**Methods:**

We performed a systematic search of all articles published up to December 2022 in the following databases: Cochrane Library, PubMed (MEDLINE) and Scopus. The meta-analysis was performed using a random-effects model. Heterogeneity was determined by the I^2^ statistics and the Cochrane Q test. The included data were analyzed to identify the proportion of indications, success rate and complications of ERCP in children.

**Results:**

Based on data from 52 studies with a total of 5624 participants, the most common indications for ERCP in children were biliary [48% (95% CI: 0.40 - 0.57; I^2^ = 98.17%, *P* < 0.001)] and both biliary and pancreatic [41% (95% CI: 0.33 - 0.49; I^2^ = 98.27%, *P* < 0.001)]. The success rate of ERCP was 95% (95% CI: 0.94 - 0.96; I^2^ = 82.53%, *P* < 0.001) with the overall complication rate of 7% (95% CI: 0.05 - 0.09; I^2^ = 82.06%, *P* < 0.001). The pooled estimate for the incidence of post ERCP pancreatitis was 4% (95% CI: 0.03 - 0.06; I^2^ = 85.46%, *P* < 0.001) and the bleeding was 0% (95% CI: 0.0 - 0.0; I^2^ = 28.21%, *P* = 0.03).

**Conclusions:**

ERCP appears to be performed safely in children with a similar success rate as in the adult population.

## Introduction

Advanced endoscopy, traditionally associated with endoscopic retrograde cholangiopancreatography (ERCP) and endoscopic ultrasound (EUS), continues to evolve as new technology and techniques become available [1, 2]. Advanced endoscopists now have a variety of capabilities, including ERCP, balloon enteroscopy, Capsule Endoscopy, EUS and transabdominal ultrasonography (TUS) and deep small bowel enteroscopy [3]. Pediatric indications differ from adults for ERCP while it is comparable in adolescents such as choledocholithiasis, liver-transplantation related disorders, and malignancy. In contrast, the main indication for ERCP in newborns was diagnostic workup of neonatal cholestasis and suspected pancreaticobiliary maljunction [4, 5]. Considering the rise in the incidence of ERCP in children, an increased risk of complications was also reported in this sub-population [6]. A few authors have expressed their opinion that ERCP could be successfully carried out in children with a similar success rate as observed in adults provided, they are performed by an experienced endoscopist in children, but the evidence is inadequate [7–9]. Considering the dearth of data, a systematic in-depth review and analysis of published literature is essential. We focus the discussion on advanced endoscopic methods that have already been developed and are more widely accepted in practice, but it is worth noting that there is an explosion of new endoscopic methods that continue to expand the frontiers of endoscopic treatment.

## Method

### Search strategy

The study protocol was developed complying the Preferred Reporting Items for Systematic Review and Meta-Analysis Protocols (PRISMA-P) checklist. An independent systematic search was implemented in the Cochrane Library, PubMed (MEDLINE), Scopus and Web of Science until April 2023 by two authors (M.H.S. and S.F). The combination of following keywords and Medical Subject Heading (MeSH) terms were used in the search strategies: [“Cholangiopancreatography, Endoscopic Retrograde” OR Endoscopic Retrograde Cholangiopancreatography OR ERCP] AND [indication OR outcome OR outcomes OR success rate OR efficacy OR patient safety OR complications OR] AND [Child OR Adolescent OR Pediatrics OR Pediatric* OR youth* OR teen* OR infant]. We also hand searched the bibliographies of retrieved reviews to find potentially relevant original articles. No language or time limits were imposed in the literature search.

### Eligibility criteria

After the elimination of duplicate records, titles and abstracts of identified papers were screened and studies meeting the following criteria were included: (1) the articles were case series, prospective/retrospective cohort studies, case-control studies, or randomized) (2) the studies enrolled pediatric/children/adolescents (aged < 18 years); and (3) the articles reported indication, success rate or complication of ERCP. Duplicate data, studies with unclear information, studies involving animals, reviews and studies whose corresponding author did not offer any feedback after several emails were excluded.

### Data extraction

A detailed full-text review was independently performed by two authors (E.Sh. and AH.H.) and the following data were abstracted using standardized pre-piloted forms: reference (first author's name and year of publication), study location, sample size, type of study, participants' characteristics (gender, age), indication, success rate and complication of procedure.

Data synthesis

The statistical analysis was conducted using STATA version 11.0 (Stata Corp, College Station, TX, USA). The pooled estimates for the indications, success rate and complications of ERCP were expressed as proportions with 95% confidence intervals (95% CI) using the random effects model, and presented visually as the Forest plot [10]. Heterogeneity was examined using the I-squared (I^2^) statistic and the Cochrane Q test in which the heterogeneity was considered significant if the I^2^ value was ≥ 50%. The significance level for heterogeneity was defined with a significance level of *P* ≤ 0.10 for Cochran Q. We assessed the presence of publication bias using the Funnel plot and the Egger’s test [11].

## Results

### Study selection

**Fig. 1:**
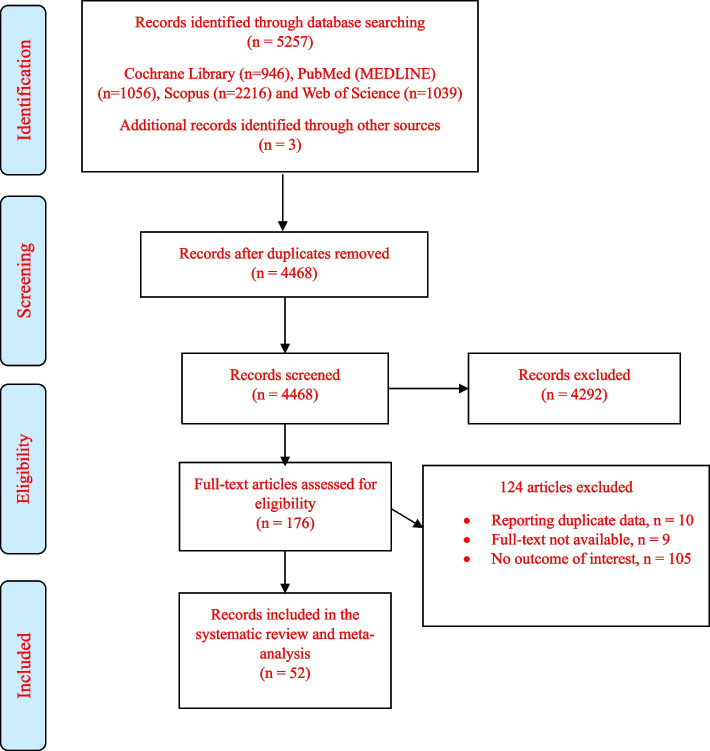
Flow chart of study selection process

Figure 1 displays the flow diagram of the study selection process. A total of 5257 articles were identified from the screening process. After the removal of duplicate records, 4467 articles remained and finally 176 articles were retained for full-text review of which 52 articles [5, 7, 9, 12–60] were included in this study.

### Study characteristics

The key characteristics of the included studies are summarized in Table 1. All except two [46, 50] had retrospective study design. In general, the included studies were published between 1993 and 2022, and were conducted in the United States of America [14, 18, 20, 21, 23–25, 29, 30, 33, 36, 40, 42, 44, 45, 48–51, 53, 57], China [17, 28, 34, 54, 56, 58–60], India [12, 16, 37], Netherlands [52], Germany, [5, 22, 32] Pakistan [46], Turkey [19, 55], Czech Republic [27], Italy [9, 35, 41], Canada [7, 38], Japan [43, 47], Korea [15], Bangladesh [39], Bulgaria [13], France [31], and Saudi Arabia [26]. The number of patients enrolled in each study ranged from 5 to 857 and the mean age (year) range of studies' participants varied between 53 days to 16.2 years. Eight studies [9, 12, 25, 28, 35, 40, 54, 59]reported exclusively on pancreatic ERCPs and three studies [27, 32, 57] on biliary ERCPs.

**Table 1:** Included study characteristics by population

**First Author (Year)**	**Study name/ study design**	**Country**	**Sample Size**	**Sex**	**Age**	**Indications**	**Success rate of diagnostic and treatment**	**Complications/ safety**
Rescorla (1995) [40]	Retrospective	USA	6	NR	4.8y	Pancreatic:6 Biliary:0 other/unknown:0	100% All six patients underwent distal	67% Two children had increased abdominal pain for 12 to 18 hours after ERCP, and one had an elevated temperature (>38°C). There were no serious adverse effects related to ERCP
Zargar (2003) [57]	Retrospective	USA	16	6 boys 10 girls	12.6y	Pancreatic:0 Biliary:7 other/unknown:9	93.8% The complete bile duct clearance was achieved in 15 patients—in the first attempt in nine patients and two attempts in six patients. In 11 patients, pus, brown muddy. One patient with gallbladder stones underwent cholecystectomy.	6% One patient developed hemorrhage immediately after ES, which resolved without blood transfusion. There were no deaths.
Prasil (2001) [38]	Retrospective	Canada	20	8 boys 12girls	11.3y	Pancreatic:5 Biliary:15 other/unknown:0	90.5% In 11 patients, the ERCP was diagnostic only, and in 10 a therapeutic procedure was done	33% 6 episodes of pancreatitis. 4 of which followed a therapeutic procedure, and 1 episode of bleeding
Green (2007) [23]	Retrospective	USA	19	6 boys 13 girls	13y	Pancreatic:4 Biliary:11 other/unknown:1	89.5% In two cases, the intended therapeutic procedure was not completed. One was the result of a CBD stone migrating and becoming lodged in the cystic duct. In the other, the bile duct could not be cannulated, presumably because a stone was impacted distally	0%
Tagge (1997) [45]	Retrospective	USA	26	11 boys 25 girls	10.1y	Pancreatic:5 Biliary:21 other/unknown:0	96% The pancreaticobiliary tree was successfully visualized by ERCP in 25 of 26 patients.	4% one death occurring in a trauma patient unrelated to his pancreaticobiliary disorder.
Paris (2010) [7]	Retrospective	Canada	29	21 girls 8 boys	10.3y	Pancreatic:29 Biliary:9 other/unknown:0	97% Only one failure to cannulate the papilla in a patient with chronic pancreatitis was encountered.	13.5% No severe pancreatitis, perforation, or bleeding was noted.
Rocca (2005) [41]	Retrospective	Italy	38	14 boys 24 girls	10y	Pancreatic:14 Biliary:24 other/unknown:0	97% Successful cannulation of the major papilla with subsequent opacification of biliary tracts or pancreatic duct was achieved in all the patients at the first attempt, except for those with extra-hepatic biliary atresia (three cases) and for one 5-week-old infant with cholestasis (final diagnosis: choledochal sludge)	6% two post-sphincterotomy bleedings (treated conservatively) and one mild pancreatitis
Teng (2000) [47]	Retrospective	Jepan	42	14 boys 28 girls	NA	Pancreatic:11 Biliary:31 other/unknown:0	100%	2% Mild cholangitis occurred as a complication in 1 patient, but was alleviated with medication.
Halvorson (2013) [24]	Retrospective	USA	45	25 boys, 20 girls	12y	Pancreatic:22 Biliary:32 other/unknown:0	97.1 % The aforementioned patient whose ERCP was aborted was a procedural failure, as well as another patient who underwent an ERCP for a pancreatic duct leak, but whose pancreatic duct disruption could not be traversed during the study	7% Complications included infection (moderate-1), bleeding (moderate-1), and post-ERCP pancreatitis (mild-1, moderate-2)
Vegting (2009) [52]	Retrospective	Netherlands	61	38 boys 23 girls	7y	Pancreatic:10 Biliary:51 other/unknown:0	71% This lower success rate was related to the relatively large group of young children with biliary atresia, in whom it is impossible to visualize the ductal system.	8% One patient experienced pancreatitis after ERCP and was treated conservatively. After 3 ERCPs in 2 different patients, pancreatic irritation occurred (abdominal pain and slight elevation of amylase). Other complications were directly related to the therapeutic interventions and included stent dislocation in 2 patients and stent occlusion in 3 patients
Issa (2007) [26]	Retrospective	Saudi Arabia	125	77 boys 48 girls	13.3y	Pancreatic:9 Biliary:115 other/unknown:0	96.8% children while cannulation of the Ampulla failed in four.	4% There was no mortality. One had bleeding from the site of sphincterotomy which stopped after adrenaline injection. Four patients (3.2%) developed transient mild pancreatitis which settled conservatively.
Troendle (2013) [48]	Retrospective	USA	65	46 girls, 19 boys	15.2y	Pancreatic:15 Biliary:50 other/unknown:0	100%	5% Adverse events included 3 episodes of mild pancreatitis, 1 episode of moderate bleeding, and 1 episode of sphincterotomy clot causing obstruction and need for repeat ERCP within 1 week.
Varadarajulu (2004) [51]	Retrospective	USA	116	NR	9.3y	Pancreatic:49 Biliary:60 other/unknown:7	97.5%	2% Complications were of mild severity and occurred only in association with grade III procedures
Agarwal (2014) [12]	Retrospective	India	172	102 boys, 70 girls	13.8y	Pancreatic:172 Biliary:0 other/unknown:0	83% 2 had no improvement in pain, and 2 underwent a surgical drainage procedure. 12 patients had pain relief.	4% Mild post-ERCP pancreatitis occurred in 2 patients (1.5%), abdominal pain with normal serum amylase/lipase levels in 6 patients (3.4%) requiring admission (*n* = 3) or prolongation of hospital stay by 2 to 3 days (*n* =3)
Dua (2008) [18]	Retrospective	USA	185	112 girls 73 boys	NA	Pancreatic:43 Biliary:71 other/unknown:71	98% In one patient, cannulation was unsuccessful and, in another, the endoscope could not be advanced into the duodenum because of altered gastric anatomy	2% Complications noted were mild pancreatitis in two and self-limited bleeding in one
Otto (2011) [33]	Prospective	USA	167	98 girls 69 boys	14.4y	Pancreatic:148 Biliary:31 other/unknown:0	72% ERCP was successful in identifying a source for recurrent pancreatitis, and nearly half of the patients with an identified anatomic abnormality went on to surgical intervention	5% Complications occurred for only 11 patients (4.76%), including 7 cases of post-ERCP pancreatitis
Saito (2014) [43]	Retrospective	Japan	220	85 boys, 135 girls	4y	Pancreatic:5 Biliary:181 other/unknown:32	96%	10% Hyperamylasemia developed in 9.4%
Limketkai (2013) [29]	Retrospective	USA	154	68 boys and 86 girls	11.5y	Pancreatic:52.2% Biliary:47.8% other/unknown:8	94.1 % due to an inability to cannulate the duct of interest: biliary or pancreatic in some patients	6% post-procedure pancreatitis (12 cases; 4.2 %), hypoxia (3; 1.0 %), and hemorrhage (2; 0.7 %)
Giefer and Kozarek (2015) [21]	Retrospective	USA	276	181 girls 95 boys	13.6y	Pancreatic:210 Biliary:194 other/unknown:11	95%	13% The most common complication was post-ERCP pancreatitis which occurred in 26 cases (7.7 %)
Enestved (2013) [20]	Retrospective	USA	296	210girls, 219boys	14.9y	Pancreatic:51 Biliary:268 other/unknown:92	95.2% 3 were deemed to be the result of an endoscopist-related factor such as inability to identify an ampulla, bleeding from a precut sphincterotomy precluding cannulation, or inability to advance beyond the pylorus because of altered anatomy. The remaining 3 cases in which cannulation was not attempted were associated with a non–endoscopist-related factor, including the inability to adequately anesthetize the patient and precipitation of a severe nasal hemorrhage during anesthesia induction	17% Post-ERCP pancreatitis occurred in 6.3% (27) of ERCPs, which included 5 cases in which patients were admitted with acute pancreatitis and the ERCP resulted in an exacerbation of their pancreatitis Abdominal pain (in the absence of fever or pancreatitis), which prolonged hospital stay, occurred after 24 (5.6%) ERCP patients.
Brown (1993) [14]	Retrospective	USA	92	60 girls 32 boys	4 months to 19 years	Pancreatic:35 Biliary:12 other/unknown:53	95.8% The most common findings included chronic pancreatitis (26 cases), pancreas divisum (14), dilated pancreatic duct (10), gallstones or sludge (8), and abnormal common bile duct (8).	15.21% 4 cases of post-ERCP pancreatitis
Perrelli (1996) [35]	Retrospective	Italy	5	3 boys, 2 girls	10.8y	Pancreatic:5 Biliary:0 other/unknown:0	100% Endoscopic pancreatic sphincterotomy, with or without removal of calculi, was performed in four cases (2 in the authors' hospital, 2 in another institution).	0% No deaths or complications occurred.
Hsu (2000) [25]	Retrospective	USA	22		10.7y	Pancreatic:22 Biliary:0 other/unknown:0	100%	6% Both patients had undergone sphincter manometry and developed mild pancreatitis. One patient had only a diagnostic manometry and the other had also undergone a biliary sphincterotomy.
Poddar (2017) [37]	Retrospective	India	72	34 girls 38 boys	8.8y	Pancreatic:28 Biliary:44 other/unknown:0	97% Of the 44 cases with suspected biliary tract disease, 14 had a choledochal cyst, 13 had portal biliopathy, two each had CBD stones, primary sclerosing cholangitis and a bile leak, one had biliary ascariasis, eight had a normal cholangiogram, and CBD cannulation failed in two. Eight of the 28 children with suspected pancreatic disorders had chronic pancreatitis, five had pancreatic duct disruption, three had pancreas divisum and the rest had a normal pancreatogram (including all eight children with unexplained abdominal pain).	8% mild exacerbation of underlying chronic pancreatitis in four, infection of a pseudocyst in one, and mild pancreatitis in one child with a choledochal cyst. The child with an infected pseudocyst (pancreatic abscess) underwent surgery.
Pfau (2002) [36]	Retrospective	USA	43	21 boys 22 girls	13.5y	Pancreatic:28 Biliary:25 other/unknown:0	94%	6% The three complications that occurred were two cases of mild post-ERCP pancreatitis and a case of postsphincterotomy bleeding.
Vrochides (2005) [53]	Retrospective	USA	100	63 girls 37 boys	16.2y	Pancreatic:26 Biliary:74 other/unknown:0	95% An intraoperative cholangiography was performed in 45 patients, and common bile duct stones were identified in 13. Expectant management of asymptomatic common bile duct stones was associated with sonographic resolution within 1 week. One patient with intraoperative cholangiography–proven choledocholithiasis required ERCP for symptoms 24 hours after operation.	0% There were no choledocholithiasis- or ERCP-related complications.
Durakbasa (2008) [19]	Retrospective	Turkey	28	15 boys 13 girls	13y	Pancreatic:7 Biliary:21 other/unknown:0	100% ERCPs were performed for biliary pathology in 21 (75%) children and for pancreatic pathology in 7 (25%). Of these procedures, 31 (97%) ERCPs were diagnostic and a therapeutic intervention was undertaken in 20 (63%) cases. A pre-cut papillotomy with a needle knife was necessary on 6 (19%) occasions because biliary cannulation was difficult.	6% the development of mild self-resolving pancreatitis in one patient and stent occlusion in another
Taj (2012) [46]	prospective	Pakistan	40	18 boys, 22 girls	13.6y	Pancreatic:19 Biliary:21 other/unknown:0	98% ERCP was successful in 51 of 52 procedures. Single procedure was performed in 36 patients, where as two patients required 2 procedures and it was repeated 4 and 6 times in the remaining two patients.	1.9% which included mild pancreatitis, whereas asymptomatic hyperamylasaemia was seen in 11% (6/52 procedures). No mortality related to ERCP occurred.
Li (2010) [28]	Retrospective	China	42	20 boys 22 girls	11.8y	Pancreatic:42 Biliary:0 other/unknown:0	91% Five patients underwent subsequent surgery because of refractory abdominal pain after endotherapy. Of the remaining 37 patients who received therapeutic ERCP alone, abdominal pain improved in 30 (81.1%) patients, and was completely relieved in 24 (64.9%) patients during the period of follow-up.	17.3% including mild and moderate pancreatitis (*n*=17) and mild cholangitis (*n*=2).
Keil (2010) [27]	Retrospective	Czech Republic.	104	48 boys, 56 girls	1.7y	Pancreatic:0 Biliary:104 other/unknown:0	91.3 Biliary atresia of any type was found in 51 children (53.7 %), with a sensitivity of 86 %, a specificity of 94 %, a PPV of 96 %, and a NPV of 100 %. Choledochal cysts were found in seven children (7.4 %), with a sensitivity of 100 %, a specificity of 90 %, PPV of 86 %, and NPV of 100 %. Biliary stones were found in seven patients (7.4 %). Other structural pathology was found in six patients, and no abnormality was seen in 24 patients.	0% No severe complications occurred during or after ERCP.
Mercier (2021) [31]	Retrospective	France	271	141 boys, 130 girls	10.9y	Pancreatic:100 Biliary:171 other/unknown:0	90%	19% 12%Post ERCP pancreatitis, sepsis 5%
Deng (2021) [17]	Retrospective	China	66	35 girls, 31 boys	NA	Pancreatic:54 Biliary:19 other/unknown:19	100%	20.7% Post ERCP pancreatitis was identified in 19 patients; there were ten mild cases, eight moderate cases, and one severe case.
Goetz (2020) [22]	Retrospective	Germany	126	56 girls, 70 boys	64 days	Pancreatic:1 Biliary:85 other/unknown:40	14.3%	Endoscopic sphincterotomy-related bleeding in 1 case
Dahale (2019) [16]	Retrospective	India	126	67 boys, 59 girls	1-15y	Pancreatic:48 Biliary:78 other/unknown:0	86% Five of these had chronic pancreatitis, four had choledocholithiasis, two had pancreatic duct leak while one had biliary leak	4.8% mild pancreatitis (2), retroperitoneal duodenal perforation (2), sphincterotomyrelated bleed (2) and hypoxia (2)
Kohoutova (2019) [9]	Retrospective	Italy	38	NR	13y	Pancreatic:38 Biliary:0 other/unknown:0	100%	3% Cholecystitis 2 cases, Bleeding 1 case
Wen (2019) [54]	Retrospective	China	38	NR	10y	Pancreatic:38 Biliary:0 other/unknown:0	100%	14.9% including pancreatitis of 13.5% and hemorrhage of 1.4%
Negm (2018) [32]	Retrospective	Germany	251	137 boys, 114 girls	53 days	Pancreatic:0 Biliary:251 other/unknown:0	89.2% The intervention failed in 27 (10.8%) infants due to duodenal stenosis (*n* = 6), pylorus stenosis (*n* = 1), small papilla (*n* = 10), or other reasons (*n* =10)	0%
Cho (2017) [15]	Retrospective	Korea	198	82 boys, 116 girls	8.7y	Pancreatic:71 Biliary:127 other/unknown:0	98.9 % Lack of procedural success was due to the inability to sedate in 1 patient and failed cannulation in 2 patients.	8.7% 5.7%Post ERCP pancreatitis, sepsis 1%, bleeding 2%
Felux (2017) [5]	Retrospective	Germany	31	15 boys, 16 girls	11y	Pancreatic:1 Biliary:20 other/unknown:10	90.7%	9.3% included four episodes of mild pancreatitis (fever, elevation of lipase, abdominal pain).
Rosen (2017) [42]	Retrospective	USA	184	124 girls, 60 boys	8y	Pancreatic:26 Biliary:141 other/unknown:17	97%	10% Post procedure pancreatitis occurred in 7% (*n* = 15), whereas hemorrhage occurred in 3% (*n* = 6), and duodenal perforation managed nonoperatively occurred in 0.4% (*n* = 1)
Yıldırım (2016) [55]	Retrospective	Turkey	48	20 girls, 28 boys	13y	Pancreatic:44 Biliary:4 other/unknown:0	70.7%	16.6 % Post ERCP pancreatitis was the most common complication occurring in 6 patients Bleeding occurred in 2 patients (3.1 %) and controlled with endoscopic management
Troendle (2015) [8]	Retrospective	USA	313	NR	12.7y	Pancreatic:63 Biliary:243 other/unknown:7	85.9%	10.9% Post ERCP pancreatitis
Zhang (2020) [59]	Retrospective	China	7	3 boys, 4 girls	6.57y	Pancreatic:7 Biliary:0 other/unknown:0	100% All seven patients were diagnosed with PPF by magnetic resonance cholangiopancreatography, and all were initially treated conservatively for a mean of 34.67 ± 22.03 d with a poor response. Among five patients who underwent ERCP, one required surgery because of intubation failure; thus, the success rate of ERCP was 80%. Two patients were successfully treated with surgery	20% Among five patients who underwent ERCP, one required surgery because of intubation failure
Shah (2020) [44]	Retrospective	USA	110		13.3y	Pancreatic:31 Biliary:55 other/unknown:24	95%	6.1% Post-ERCP pancreatitis occurred in 5.2% pediatric patients. Out of 12/232 patients developing PEP in pediatric age group, 3 (1.2%) had severe pancreatitis whereas 9 (3.8%) had mild pancreatitis. No need for surgical intervention and no mortality was observed. Postsphincterotomy bleeding occurred in 1.4% in pediatric patients
Zeng (2019) [58]	Retrospective	China	75	23 boys, 52 girls	6y	Pancreatic:47 Biliary:0 other/unknown:28	100% A total of 112 ERCP procedures were performed on 75 patients with PBM (range: 1 to 5 times per patient), and the technical success rate was 100%.	16% Procedure-related complications were observed in 12 patients and included post-ERCP pancreatitis (9/75, 12.0%), gastrointestinal bleeding (1/75, 1.3%), and infection (2/75, 2.7%).
Asenov (2019) [13]	Retrospective	Bulgaria	24	7boys, 17 girls	15y	Pancreatic:2 Biliary:10 other/unknown:12	100% In 17 (71%) patients, the procedure was used for therapeutic purposes. The indications were choledocholithiasis (10 cases, 42%), postoperative complications (5 patients, 21%), and recurrent pancreatitis (2 cases, 8%).	4% There were no major complications in this series. In only 1 patient, the elevation of amylase and WBC and complaints of abdominal pain were detected.
Lin (2021) [30]	Retrospective	USA	27	14 boys, 13 girls	9.7y	Pancreatic:0 Biliary:0 other/unknown:27	100%	21% PostERCP pancreatitis (PEP)
Yu (2022) [56]	Retrospective	China	127	54 boys, 73 girls	14y	Pancreatic:13 Biliary:107 other/unknown:0	98.3%	12.2% PostERCP pancreatitis (PEP)
Rashid (2022) [39]	Retrospective	Bangladesh	20	13 boys, 7 girls	10y	Pancreatic:9 Biliary:11 other/unknown:0	93.3% Two successive attempts within a fortnight for a 10-year-old female patient with chronic pancreatitis (CP) failed	0%
Troendle (2022) [50]	Prospective	USA	857	NR	13.5y	Pancreatic:27.6% Biliary:76.4% other/unknown:0	90.5%	8% The most commonly identified adverse events included post-ERCP pancreatitis (5%), pain not related to post-ERCP pancreatitis (1.8%), and bleeding (1.2%)
Weng (2021) [60]	Retrospective	China	15	7 boys, 8 girls	10.4y	Pancreatic:5 Biliary:10 other/unknown:0	100%	11.8% post-ERCP pancreatitis
Pan (2021) [34]	Retrospective	China	46	24 boys, 22 girls	28.5 mon	Pancreatic:0 Biliary:0 other/unknown:46	87%	7.9% post-ERCP pancreatitis

### Meta-analysis

**Fig. 2:**
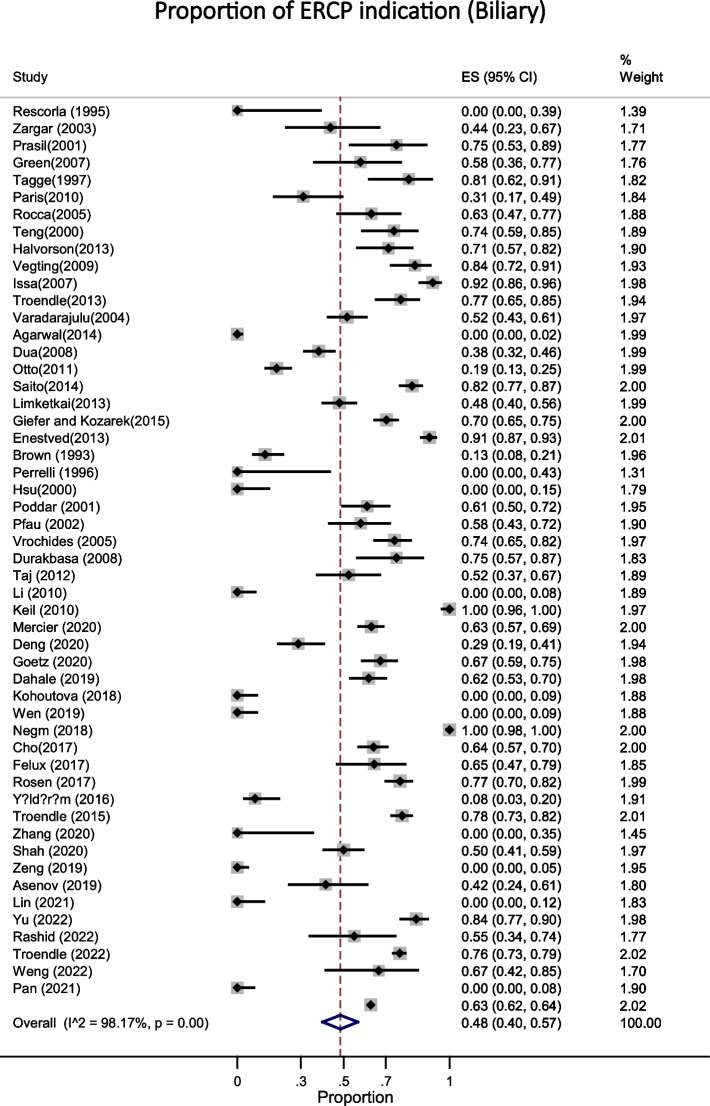
This figure shows the pooled estimate of the proportion ERCP indication (biliary) in pediatric

**Fig. 3:**
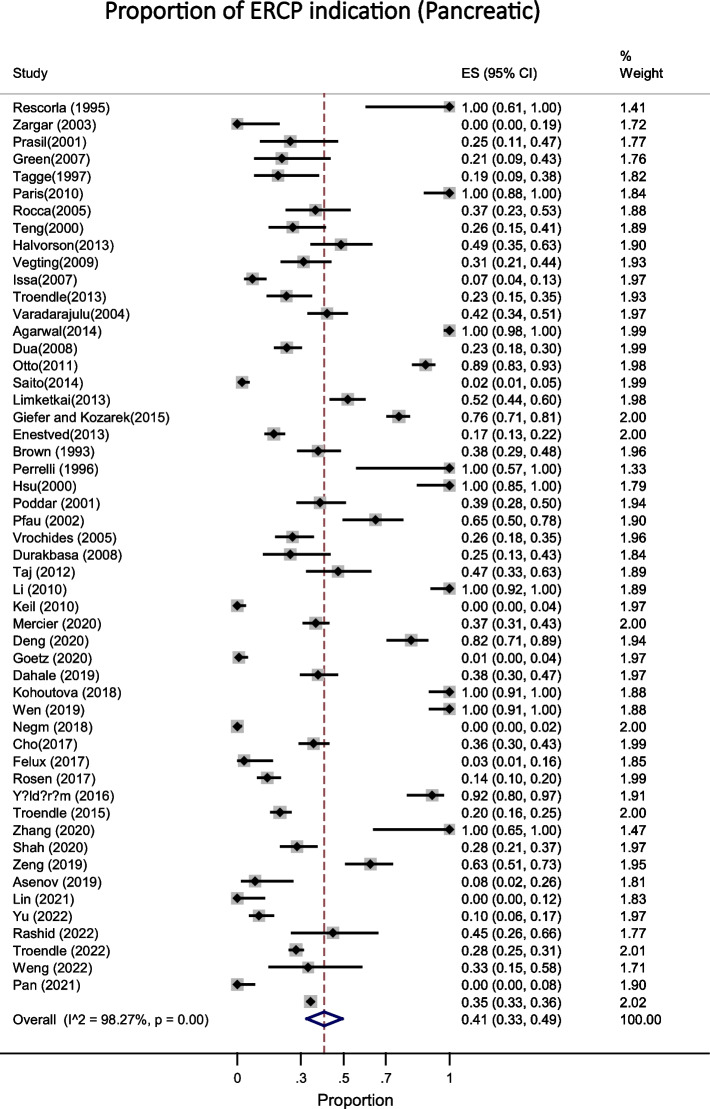
This figure shows the pooled estimate of the proportion ERCP indication (pancreatic) in pediatric

**Fig. 4:**
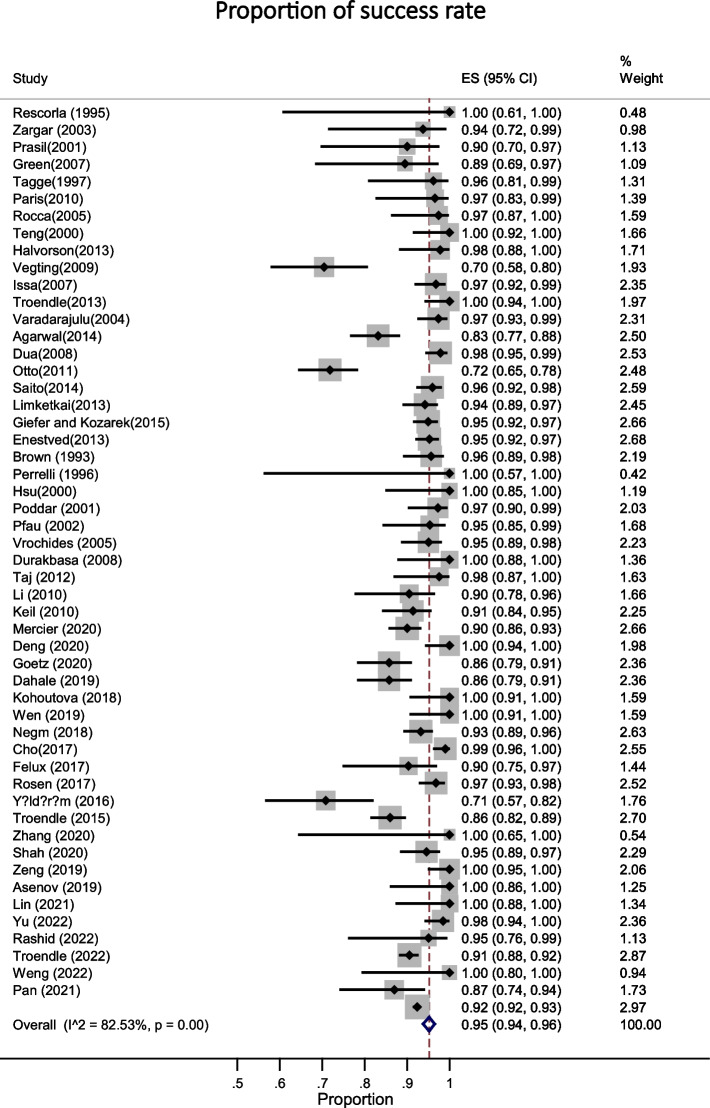
This figure shows the pooled estimate of the proportion of overall success rate of ERCP in pediatric

**Fig. 5:**
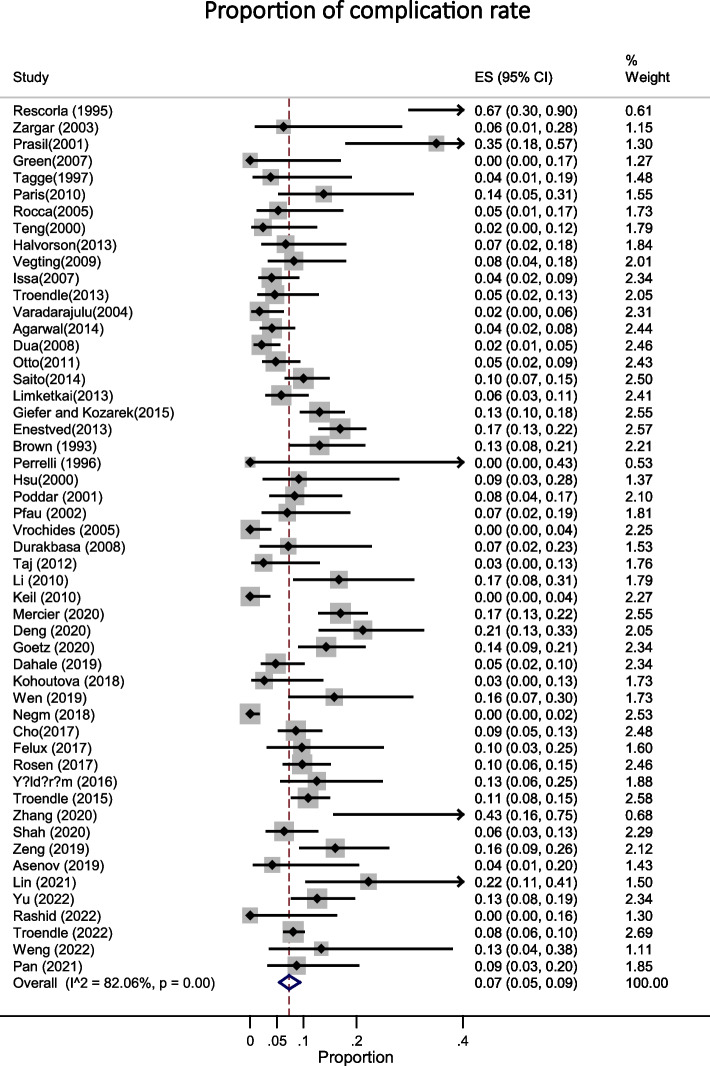
This figure shows the pooled estimate of the proportion of overall complication rate of ERCP in pediatric

**Fig. 6:**
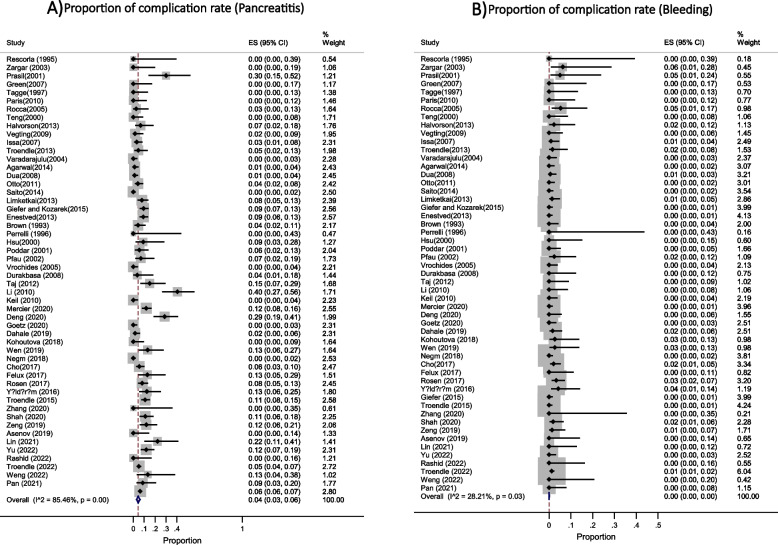
This figure shows the pooled estimate of the proportion of **A**) Post ERCP pancreatitis and **B**) Post ERCP bleeding in children

Based on the data from 52 studies with a total of 5624 participants, the most common indications for ERCP in pediatric were biliary [48% (95% CI: 0.40 - 0.57; I^2^ = 98.17%, *P* < 0.001)] in 3653 patients (Fig. 2) and both biliary and pancreatic [41% (95% CI: 0.33 - 0.49; I^2^ = 98.27%, *P* < 0.001)] in 2018 patients (Fig. 3). The success rate of the procedure as 95% (95% CI: 0.94 - 0.96; I^2^ = 82.53%, *P* < 0.001) (Fig. 4) with the overall complication rate of 7% (95% CI: 0.05 - 0.09; I^2^ = 82.06%, *P* < 0.001) (Fig. 5). To clarify the details of most common complication of ERCP and the source of heterogeneity, we carried out a subgroup analysis based on the Post ERCP pancreatitis and bleeding. Complication of ERCP (pancreatitis and biliary) was observed as a possible source of heterogeneity on the overall effect size. Post ERCP pancreatitis was reported 4% (95% CI: 0.03 - 0.06; I^2^ = 85.46%, *P* < 0.001) in 350 and bleeding was 0% (95% CI: 0.0 - 0.0; I^2^ = 28.21%, *P* = 0.03) as observed only in 40 patients out of the total 5900 (Fig. 6). Considering the significant heterogeneity to identify the source, further subgroup analyses were performed by age group and the ERCP center. On the basis of subgroup analyses, none of the variables examined represented a source of heterogeneity, but the subgroup analysis showed the overall complication of ERCP was greater 10% [95% CI: 0.06 - 0.14] in children between age 2-10 years compared with the other age groups [ for < 2 years: 8% (95% CI: 0.02 - 0.19) and for > 10 years: 6% (95% CI: 0.05 - 0.09)]. These results were similar for success rate, in that the success rate for the children between age 2-10 years was %97 (95% CI: 0.93 - 1.0) and was higher compared to other age groups [for < 2 years: 0.93 (95% CI: 0.89 - 0.96) and for > 10 years: %95 (95% CI: 0.92 - 0.96)].

### Publication bias

**Fig. 7:**
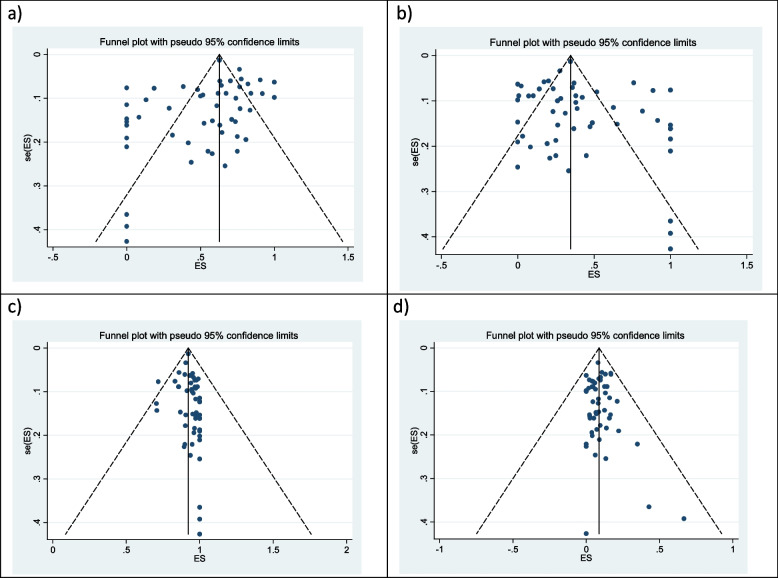
Funnel plots of primary and secondary outcomes using random-effects model **a**) biliary indication, **b** pancreatic indication, **c** success rate and **d**) complication rate

The evaluation of publication bias by visual inspection of the funnel plot and Egger’s test demonstrated some evidence for publication bias in the meta-analysis of biliary indication of ERCP (*P* = 0.040). However, the results of the meta trim and fill analysis did not reveal any presence of additional studies other than those included in this meta-analysis. Egger’s linear regression test for elevated pancreatic indication (*P* = 0.284), success rate (*P* = 0.355), and complication rate (*P* = 0.500) did not reveal presence of any publication bias (Fig. 7).

## Discussion

The present meta-analysis was carried out for evaluating the indications, success rate, and complications of ERCP in children. We observed through a total of 5624 participants in 52 studies, the most common indications were biliary and almost 95% of the procedure was successful with a very satisfactory rate of overall complication (7%). Our results are very assuring considering the fact that ERCP provides an opportunity for visualization of the biliary tract in infants and can replace the non-invasive imaging by magnetic resonance cholangiopancreatography (MRCP) [61]. Our study had a much higher success rate compared to the results carried out in adults where the pooled estimates for single-balloon enteroscopy (SBE-) assisted ERCP in biliary interventions revealed with a success rate of 75.8% [62]. Similarly, results from Sun et al. [63], revealed a success rate of 74% and the incidence of side effects as 8% compared to the present study where the success rate and complication rate of 95% and 7%, respectively. Additionally, the previous study was also limited in not revealing the indications for ERCP, unlike the present study, instead, there were interesting results regarding stent placement as the most common method (75%) and the usage proportion of sphincterotomy (ST), stone extraction/removal and bougienage/balloon dilation. Usatin *et al* [64] evaluated the same outcome measures in a meta-analysis of 32 studies (2612 study participants) where biliary indications contributed to 54% of the cases with an overall complication rate of 6%. The authors have also observed a similar rate of pancreatitis (3%) and bleeding (0.6%) as shown in the present study. Although some studies have observed injection of contrast medium in the pancreatic duct and pancreatic sphincterotomy as the risk factors for post-ERCP pancreatitis [8], we could not evaluate it in the present study owing to data constraints. Additionally, none of the studies have evaluated mortality following ERCP despite 0.11% observed in the adult population [6]. Although the overall complication rates following ERCP were low, it is still significant particularly in terms of the morbidity due to post-ERCP pancreatitis. Due to the limited number of pediatric cases requiring ERCP, in many centers, ERCP is carried out by adult gastroenterologists with a reasonable success rate and low complication rates [24]. It is still debatable whether a pediatric gastroenterologist should only perform the ERCP in children or a trained adult gastroenterologist in the pediatric procedures can be involved in doing so particularly with the limited data in the literature. Post-ERCP pancreatitis has been observed to be 4% in the present study like the rates observed in adult populations [65, 66]. We could not evaluate the complication rate in infants separately although a recent unpublished report in this sub-population revealed a higher incidence (13%) compared to older children [67].

The present meta-analysis has included the maximum number of studies and patients to date. However, the study does have certain limitations. Firstly, there is a varied length of follow-up among participants in the studies, which may lead to the observation of additional complications not documented in long-term follow-up. Additionally, the rate of observed complications may also differ between studies.

Secondly, differences in resources among hospital set-ups may result in variations in the experiences of gastroenterologists who performed ERCP between studies. This could potentially impact the outcomes and conclusions drawn from the analysis. Furthermore, there is a limited number of published studies involving younger children and infants, making it unclear if success rates and complications differ in this age group compared to older children. This lack of data raises questions about generalizability and applicability to pediatric populations. Lastly, none of the included studies have mentioned mortality following ERCP, either immediately after the procedure or due to long-term complications. This information is crucial for a comprehensive understanding of the risks associated with ERCP. Overall, while this meta-analysis provides valuable insights into ERCP outcomes based on existing literature, these limitations should be taken into consideration when interpreting the results and applying them to clinical practice.

## Conclusion

ERCP appears to be performed safely in children with similar success rates as in the adult population.

## Data Availability

Data is available upon request from the corresponding author for the article due to privacy / ethical restrictions.
